# A Case Report of Aggressive Angiomyxoma in Pregnancy: Do Hormones Play a Role?

**DOI:** 10.1155/2016/6810368

**Published:** 2016-11-15

**Authors:** Theofano Orfanelli, Chi-Son Kim, Sally F. Vitez, James Van Gurp, Neeti Misra

**Affiliations:** ^1^Department of Obstetrics, Gynecology and Reproductive Sciences, Rutgers-Robert Wood Johnson Medical School, 125 Paterson Street, New Brunswick, NJ 08901, USA; ^2^Department of Pathology, Rutgers-Robert Wood Johnson Medical School, One Robert Wood Johnson Place, MEB 212, New Brunswick, NJ 08903, USA

## Abstract

Aggressive angiomyxoma is a rare, locally invasive tumor that generally affects the perineum and pelvis of reproductive age females. Aggressive angiomyxoma is often misdiagnosed, resulting in the delay of the treatment. Case reports show increased growth of the tumor during pregnancy, thus suggesting a hormonal dependency. We report this rare condition in a 29-year-old primigravid female with a growing mass on the right labium majus at 20 weeks' gestation. The patient also developed a smaller mass on the left labium majus at 37 weeks' gestation. The patient underwent a primary cesarean section with resection of the right labial mass, with a final diagnosis of aggressive angiomyxoma. The lesion on her left labium majus resolved spontaneously postpartum. This case report supports a hormonal involvement in this tumor.

## 1. Introduction

Aggressive angiomyxoma (AA) was first described in 1983 by Steeper and Rosai [1]. It is a rare, soft tissue mesenchymal tumor, usually found in individuals of reproductive age, with female to male ratio of 6.6 : 1 [2, 3]. There have been fewer than 250 cases of women with AA reported in the world literature to date [4]. The local recurrence rate of AA after surgical excision varies from 25% to 47% [5]. Recurrences may occur from months to several years after excision (2 months to 20 years) [4, 5]. There have only been three reported cases of AA metastasis [6–8]. The primary management has been wide local tumor excision [2–4]. However, expression of estrogen and progesterone receptors in AA suggests that tumor growth is hormonally driven and thus hormonal modulation and suppression could be an alternative, noninvasive treatment [9]. To our knowledge there are only 15 cases of AA in pregnancy reported in the literature. We report our experience with a pregnant patient found to have an AA and the progression of her disease.

## 2. Case Presentation

**Figure 1:**
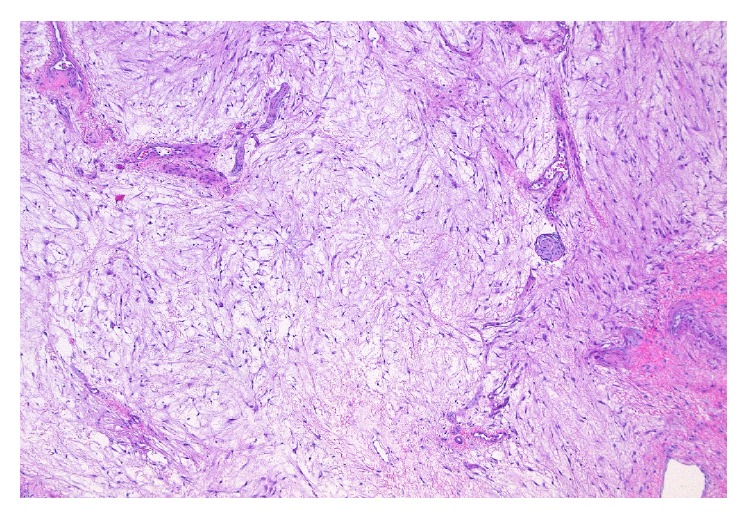
Tissue section from the right labial mass showing tumor composed of spindle- and stellate-shaped cells in a myxoid matrix, hematoxylin and eosin 400x.

**Figure 2:**
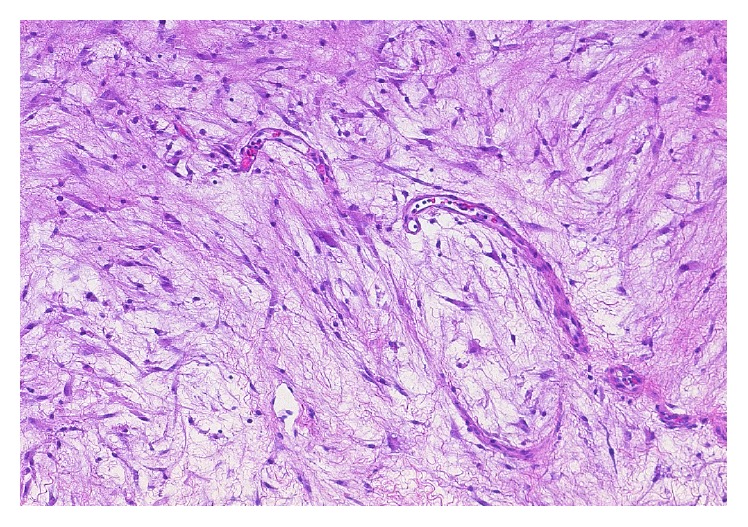
Tissue section from the right labial mass showing tumor composed of capillary spindle-shaped channels, hematoxylin and eosin 1000x.

A 29-year-old primigravid female with an uncomplicated pregnancy and unremarkable medical and surgical history presented at 20 weeks' gestation complaining of new onset painless, right labial swelling. On examination there was a 2 × 2 cm polypoidal mobile nontender edematous mass on the right labium majus. No lesions were appreciated in the left labium majus. Condyloma acuminatum was suspected and the decision was made to monitor her. However, the mass continued to grow throughout the pregnancy, causing significant discomfort. By 37 weeks of gestation the mass measured 7 cm in length from the perineum obstructing the vaginal canal. At the same time, the patient also reported similar, painless swelling of her left labia. On examination, a discrete mass, less than 1 cm, was noticed in the left labium majus. A biopsy of the right labial lesion was recommended and plan for cesarean section to decrease the risk of birth trauma was discussed with the patient. Patient declined the biopsy as an outpatient expressing concerns about possible discomfort related to the procedure with local anesthesia and opted for excision of the right labial lesion at the time of the cesarean section. The patient underwent elective primary cesarean section with spinal anesthesia at 39 weeks' gestation due to increased risk of birth trauma and bleeding from the right labial lesion with a vaginal delivery. In addition, excision of the right labial mass was performed, which measured 4.5 cm at the time of surgery. Given the small size of the left labial lesion (<1 cm) at the time of the delivery and in order to minimize the risks from multiple invasive procedures, the sampling of the left labial lesion was deferred. Macroscopic description of the specimen received by pathology is as follows: polypoid fragment of edematous, lobulated, wrinkled tan skin; at the base of the specimen there is area of edematous stalk-like tissue; longitudinal section through the polypoid mass reveals marked edematous “botryoid” cut surface. The histology of the right labial mass confirmed the diagnosis of AA extending into the surgical margins (Figures 1 and 2), with immunohistochemical staining positive for CD34, estrogen, and progesterone receptors. The postoperative course was uncomplicated. The left labium majus mass spontaneously regressed in size within 2 weeks postpartum. The patient was seen by a gynecologic oncologist 4 weeks postpartum, at which point she no longer had complaints of labial swelling and only a 0.5 cm edematous lesion was identified at the edge of the left labium minus. The patient was counseled on excision of the left labial lesion, with possible reexcision of the right side to obtain margins, versus conservative observation every 2-3 months. The patient opted for conservative management and there has been no tumor recurrence for the last 20 months since the diagnosis.

## 3. Discussion

Aggressive angiomyxoma (AA) is a slow growing, locally invasive tumor. Clinically, patients present with incidental finding of a nontender mass of different sizes. The patients are often otherwise without other complaints. Due to the rarity of this disease and the clinical presentation, it is often misdiagnosed as condyloma acuminata, Bartholin duct cysts, lipoma, vulvar abscess, Gartner duct cyst, vaginal cyst, vaginal prolapse, levator hernia, or sarcoma. Fibroepithelial stromal polyp, superficial angiomyxoma, angiomyofibroblastoma, cellular angiofibroma, and smooth muscle tumors also need to be considered in the differential diagnoses of a polypoidal mass in the perineum [10]. The pathogenesis of this disease is still unclear. Recent findings demonstrate a translocation at chromosome 12, with a consequent aberrant expression of the high-mobility group protein isoform I-C (HMGIC) protein involved in DNA transcription. Detection of this inappropriate expression may potentially be used as a marker for microscopic residual disease [11].

Histologically, AA is composed of a mixture of spindle and stellate cells in myxomatous matrix. What is distinct is the prominent vascular component with large thick-walled vessels without anastomosis or arborization. This differentiation helps to distinguish AA from other soft tissue tumors. Immunohistochemistry is positive for vimentin, desmin, smooth muscle actin, and, less commonly, estrogen and progesterone [12].

Imaging modalities may assist in determining the extent of the disease and aid in surgical planning. Sonography reveals AA as hypoechoic or cystic [13]. AA attenuates less than muscles on a computed tomographic (CT) scan. On magnetic resonance imaging (MRI), it shows up with a swirled pattern. MRI has been the preferred mode of recurrence detection [14, 15]. Our patient was not subjected to radiological investigation, as its clinical appearance at presentation was that of a benign lesion.

Although wide surgical margins have been shown to have no effect on prognosis when compared to narrow margins, the primary mode of treatment remains the wide local excision and is often extensive due to the ill-defined aspect of the tumor. Complete excision may involve removal of the adjacent fascia and muscles. Cases with involvement of the genital tract, bladder, gastrointestinal tract, and bone have been described in the literature requiring extensive surgical resection to achieve clears margins, however, associated with a major impact on fertility and significant perioperative morbidity [16].

Emerging therapies to minimize mutilating surgery include hormonal therapy, angiographic embolization of the mass, and radiotherapy. GnRH agonists have been used to treat recurrent AA, primary treatment of small AA, and as adjuvant therapy. Other hormonal treatments have been tried, including tamoxifen and raloxifene, in small number of cases [17–19]. Angiographic embolization has been utilized in certain cases to shrink the mass by devascularization the tumor. However, recurrence after angiographic treatment may ensue due to development of an alternative blood supply to the tumor [20]. This modality may be considered for AA needing extensive excision when preservation of fertility is desired or high-operative morbidity due to extensive surgery is anticipated. Radiation has been used in rare cases of recurrent, nonsurgical candidates. AA with low mitotic index may have limited response to radiation therapy. There is no evidence that adjuvant radiation therapy lowers the recurrence rate. However, it should be considered in patients with no response to embolization or hormonal treatment and in whom mutilating surgery for tumor resection is not warranted [9, 21].

Very few case reports are available to clearly define the behavior of AA in pregnancy. The presence of hormonal receptors in AA developed during pregnancy supports a hormonal dependency [9, 22–24]. Fishman and Otey described the first case in 1995, a 37-year-old woman who was not pregnant at the time of initial AA diagnosis. The tumor was resected and was found to express both estrogen and progesterone receptors. Six months later, in the second trimester of her pregnancy, she was diagnosed with tumor recurrence. Ultimately, the patient had a normal vaginal delivery. Reexcision was advised but she was lost to follow-up. She presented three years later with a large labial mass and underwent surgical excision at that time [22]. Han-Geurts et al. reported three cases of women aged 31, 27, and 34, who presented with a primary AA found during pregnancy in the pelvis, the right abdomen, and the right labium majus, respectively. The first two patients were managed with exenteration after delivery. The patient with the pelvic tumor also received adjuvant radiation therapy. Immunohistochemical staining was not reported for these cases [9]. Bagga et al. reported a 25-year-old woman at 12 weeks' gestation with swelling in the right labium majus. The mass was excised at 16 weeks' gestation. The diagnosis was consistent with AA, with immunohistochemical staining positive for estrogen and negative for progesterone receptors. The patient had no recurrences at evaluation 9 months later [23]. The first case to closely monitor AA growth before, during, and after pregnancy was described by Aye et al. A 22-year-old woman with known AA status after neoadjuvant GnRH analogue therapy and excision of the lesion in the region of the right Bartholin's gland was found to be pregnant. At 20 weeks' gestation, a mass in the area of the original AA was noted to gradually increase in size. Elective cesarean section was performed, and an MRI 6 weeks postpartum showed that the lesion had reduced in size without any treatment [24].

## 4. Conclusion

Our case presents a rare disease found in pregnancy. The findings of rapid growth during pregnancy, with spontaneous regression in the postpartum period, support the theory of hormone driven tumor growth. Primary care physicians, in addition to obstetricians and gynecologists, should maintain a high index of suspicion for patients presenting with enlarging vulvar or perineal lesions, especially in pregnancy, as this may be the first and only symptom of an insidious process. Due to its high recurrence rate, all patients should be counseled about the importance of long-term follow-up care. Although surgical wide local excision has been the primary treatment modality, medical treatment with or without narrow margin excision can be considered based on the data available. Further research into multi-interdisciplinary treatment may decrease the risk of recurrence and prevent the need for extensive wide local excision in the young population primarily affected with AA.
